# Maize Fungal Growth Control with Scopoletin of Cassava Roots Produced in Benin

**DOI:** 10.1155/2017/5671942

**Published:** 2017-01-18

**Authors:** Rafiatou Ba, Teou Alfa, Fernand Gbaguidi, Kosi Mawuéna Novidzro, Kokouvi Dotse, Koffi Koudouvo, Ursula Houngue, Marcel T. Donou Hounsode, Kossi Honoré Koumaglo, Yaovi Ameyapoh, Lamine Baba-Moussa

**Affiliations:** ^1^Laboratoire de Biologie et de Typage Moléculaire en Microbiologie, Département de Biochimie et de Biologie Cellulaire/FAST/UAC, Cotonou, Benin; ^2^Laboratoire de Pharmacognosie et des Huiles Essentielles/CBRST, Porto-Novo, Benin; ^3^Laboratoire de Microbiologie et de Contrôle de Qualité des Denrées Aliments, Ecole Supérieur des Techniques Biologiques et Alimentaires, Université de Lomé, Lomé, Togo; ^4^Laboratoire des Extraits Végétaux et Arômes Naturels (LEVAN), Département de Chimie, Université de Lomé, 01 BP 1515 Lomé, Togo; ^5^Laboratoire de Biomathématiques et d'Estimations Forestières, Faculté des Sciences Agronomiques, Université d'Abomey-Calavi, Abomey-Calavi, Benin

## Abstract

The chemical contamination of food is among the main public health issues in developing countries. With a view to find new natural bioactive products against fungi responsible for chemical contamination of staple food such as maize, the antifungal activity tests of scopoletin extracted from different components of the cassava root produced in Benin were carried out. The dosage of scopoletin from parts of the root (first skin, second skin, whole root, and flesh) was done by High Performance Liquid Chromatography. The scopoletin extract was used to assess the activity of 12 strains (11 strains of maize and a reference strain). The presence of scopoletin was revealed in all components of the cassava root. Scopoletin extracted from the first skin cassava root was the most active both as inhibition of sporulation (52.29 to 87.91%) and the mycelial growth (36.51–80.41%). Scopoletin extract from the cassava root skins showed significant inhibitory activity on the tested strains with fungicide concentration (MFC) between 0.0125 mg/mL and 0.1 mg/mL. The antifungal scopoletin extracted from the cassava root skins may be well beneficial for the fungal control of the storage of maize.

## 1. Introduction

Cereals are fundamental staple food in developing countries especially for rural populations of low revenues [1]. Among cereals, maize is considered as the main agricultural sector and therefore has an important political support in West Africa [2]. Maize is one of the most important cereal grain crops worldwide, together with rice and wheat, and it is an important forage crop [3]. Its added value is more than two billion euros, mainly helps rural people, and provides more than 10 million permanent jobs [4]. In Benin, maize is grown nationwide and thus ranking first among cereals such as sorghum and millet [5]. The increase in maize production draws the attention of farmers to its storage and conservation. The problems faced by maize producers during postharvest have long been overlooked [6]. Adégbola et al. [7] reported that inadequate storage facilities are often the cause of these postharvest losses. In effect, every year, producers record postharvest losses ranging from 20 to 50% after six (06) months of storage [8]. It was observed that women recorded postharvest losses up to 75% when they failed to apply any phytosanitary treatment [9]. Besides, Borbély et al. [10] have examined mycotoxin levels in cereal samples and mixed feed samples collected in eastern Hungary and detected aflatoxin B1 levels above the EU limit in 4.8% of the samples. Recently, Dobolyi et al. [11] identified aflatoxin producing* A. flavus* from maize kernel collected in various parts of Hungary. Furthermore, Tóth et al. [3] have examined the occurrence of potential mycotoxigenic fungi on Hungarian maize kernels between 2010 and 2012. Chemical contamination caused by toxigenic fungi of staple foods such as maize is a critical issue for public health. Maize kernels are often infected with fungi that synthesize mycotoxins, which pose major threats to human and animal health [12, 13]. Mycotoxins are secondary metabolites of filamentous fungi, which are harmful to animals and humans, and are able to induce various disease symptoms [14–16]. Although direct evidence that fumonisins cause serious human and animal health problems is lacking, human esophageal cancer is reportedly associated with the consumption of maize-based products contaminated with fumonisins [17]. In warmer regions,* Fusarium* ear rot is more prevalent; in this disease, kernels are infected by* Fusarium verticillioides* or other species of the* Gibberella fujikuroi* complex, including* F. proliferatum *and* F. subglutinans* [18]. Over the last decade, the most commonly isolated pathogen worldwide has been* F. verticillioides* Leslie and Summerell [19]; the fungus has been identified in Spain [18]; Brazil [20]; Canada [21]; Nepal [22]; and Nigeria [23]. Chemical control remains the primary measure to reduce the incidence of postharvest contamination [24]. Similarly, the restriction imposed by the food industry and regulatory agencies on the use of some synthetic food additives has led to renewed interest in the search for alternatives, such as natural antimicrobial compounds, especially those of vegetable origin [25]. Coumaric compound, belonging to the groups of phytoalexins, is the active ingredient present in several plants' scopoletin [26] and was therefore the subject of several studies. But, for the best of our knowledge, very few investigations have been conducted on the use of scopoletin extracted from different parts of cassava root to control the growth of maize mold strains in their storage. Herein, we aim to assess the antifungal activity of scopoletin extracted from cassava root parts on mold strains isolated from maize in storage.

## 2. Materials and Methods

### 2.1. Plant Material

The plant material consists of cassava roots of variety BEN 86052 harvested from the fields of the National Institute of Agricultural Research of Benin situated at Abomey-Calavi Township. The cassava root samples were dried in the sun on grid at room temperature to obtain cassava chips.

### 2.2. Microbiological Materials

Microbiological materials comprise 12 strains including 11 isolated from the maize in storage and a reference strain (*A. parasiticus *CMBB20) (Table 1). The reference strain was provided by the Hygiene Section of Water and Food of the Benin National Public Health Laboratory. The identification of fungal species was based on macroscopic observation (mycelium morphology, growth rate, color, and texture of thallus) and microscopic examination of a fungal colony [27–29].

### 2.3. Extraction and Dosage of Scopoletin of Cassava Roots

**Figure 1:**
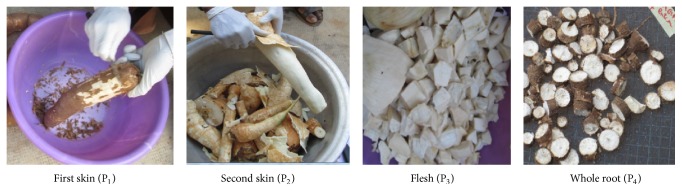
Different parts of cassava root.

**Figure 2:**
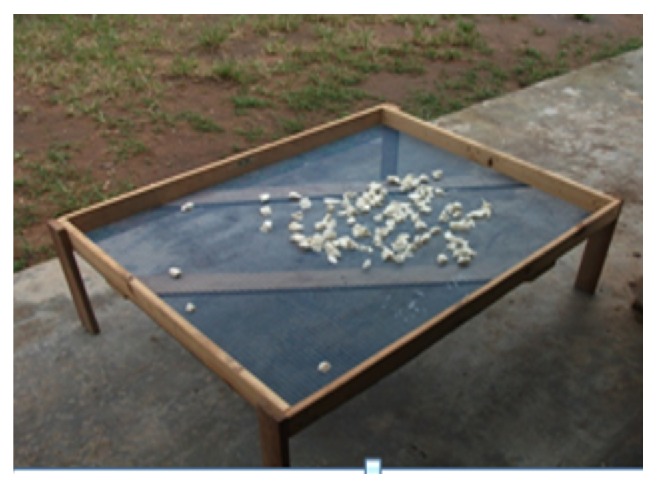
Drying parts of cassava root in the sun on grid at room temperature (28°C ± 2).

Scopoletin was extracted from each part of cassava roots. It includes the first skin (P_1_); the second skin (P_2_); the flesh (P_3_); and the whole root (P_4_) (Figure 1). These different parts were dried in the sun on grid at room temperature for six days to obtain cassava chips (Figure 2). After drying, the skin, the flesh, and the whole root samples were ground with an electric mill. Scopoletin was extracted with absolute ethanol as described by Buschmann et al. [30]. For each part of the cassava root, 10 mL of absolute ethanol was added to 2 g and 4 g of powder, respectively. After 3 days of maceration, the resulting mixture was then filtered and concentrated to a final volume of 3 mL. Scopoletin was then assayed on a high performance liquid chromatograph in the analytical conditions described by the method developed by Ba et al. [31].

### 2.4. Qualitative Assessment of Scopoletin in Cassava Root

The presence of scopoletin in the various components of the cassava root was assessed by thin layer chromatography as described by the French Pharmacopoeia [32]. The extracts obtained above were deposited on a grafted silica gel plate RP 18. The mixture Toluene-Ethyl Acetate (15; 85) was used as eluent (mobile phase). After elution the plate was observed at 356 nm under UV. Pure scopoletin (Sigma®, S2500-50MG) was used as standard and the spots of the various extracts were compared to that of the reference scopoletin.

### 2.5. Assessment of the In Vitro Antifungal Activity of Scopoletin on Fungal Strains Isolated

The antifungal activity of scopoletin extracted from cassava roots was investigated in vitro and determining the Minimum Inhibitory Concentration (MIC) of the extracts was aimed. These tests were carried out on a solid medium and consisted primarily of the realization of antifungal screening and the determination of Minimum Fungistatic Concentration (MFC) and Minimum Fungicidal or Lethal Concentrations (MLC).

*Antifungal Screening*. The antifungal screening was carried out following the method described by Dohou et al. [33]. This method permits to identify and select efficient plant extracts against fungi. Scopoletin activity was evaluated on eleven (11) strains isolated from maize in storage and the reference strain* A. parasiticus* (CMBB20). Two stages of fungal growth (mycelial growth and sporulation) were observed. Powders of different parts of the cassava root dissolved in absolute ethanol were tested at concentrations of 0.2 mg/mL and 0.4 mg/mL. The extracts thus obtained were mixed with hot Potato Dextrose Agar (PDA) (50°C) and 10 mL of each mixture (extract-PDA) was poured into Petri dishes. After the polymerization of agar, a fungal inoculum volume equivalent to 100 spores was deposited in the middle of each Petri dish. The fluconazole (0.1 mg/mL) and the PDA culture medium (without extract) were used as positive and negative control, respectively. The tests were performed in triplicate and the plates were incubated at 28°C ± 2. After 5 days of incubation, the mycelium diameter was measured each on test plates and on control plates. With the aid of a Malassez cell (Germany; P 0.200 mm; S 0.0025 mm^2^), the number of spores was determined microscopically (microscope XSZ-107BN). The percentages of inhibition of mycelial growth and the sporulation of the extracts were calculated relative to a negative control (plate without extract) using the formula below:

(1) $$\begin{array}{c} \text{PI}=\frac{\left(A-B\right)}{A}\times 100. \end{array}$$

See [34].

PI is % of inhibition, *A* is average diameter of the mycelium or the average number of spores on control medium, and *B* is average diameter of the mycelium or the average number of spores in the presence of the tested extract.

When the percentage of inhibition is between 75 and 100%, the extract is very active and the fungal strain is considered very sensitive. When this percentage is between 50 and 75%, the extract is regarded as active and the fungal strain sensitive. Between 25 and 50%, the extract is moderately active and the strain is called limit; the extract is either little or nonactive when the percent inhibition is between 0 and 25%; in this case the strain is considered insensitive or resistant [35].

### 2.6. Determination of Minimum Inhibitory Concentrations

Minimum Inhibitory Concentrations (MIC) and Minimum Fungicidal or Fungistatic Concentrations (MFC) were determined using microdilution method with Sabouraud liquid broth [36]. Each fungal inoculum was obtained from a suspension in physiological water of spores from a culture of seven days on PDA. Fungal suspensions were distributed in 96-well plates at 100 *μ*L per well. The increasing concentrations of pure scopoletin and extracts of scopoletin (scopoletin from the first skin, second skin, and the mixture of the two skins) were obtained by serial dilution (1/2 to 1/64) of a stock solution 0.4 mg/mL. Thus, the corresponding concentrations 0.2 mg/mL; 0.1 mg/mL; 0.05 mg/mL; 0.025 mg/mL; 0.0125 mg/mL; and 0.00625 mg/mL were tested by their addition to the culture broth. A fungal inoculum volume corresponding to opacity of 5 to 10^5^ spores was determined by counting the spores in a Malassez cell (Germany; P 0.200 mm; S 0.0025 mm^2^) from control wells without extract (negative control). The microplates were covered with wax paper and incubated at 28°C ± 2 for 72 h. Two other controls were also performed, that is, the positive control (broth plus suspension) and ethanol control (ethanol plus suspension). The MIC corresponds to the lowest concentration at which no fungal growth was observed with the naked eye. To the experimental concentrations where no growth or sprouting was observed, Fungicidal or Fungistatic activity was tested (Boniface et al. [37]). The Minimum Fungicidal Concentration is defined as the minimum concentration that kills 99.99% of the initial inoculum. The MFC was determined by taking 100 *μ*L of suspension in the wells without growth on nutrient agar PDA. PDA plates were incubated at 28 ± 2°C for 5 days. The ratios MFC/MIC were determined and were used to determine the type of effect produced by extracts on the fungi tested. Thus, the effect of the extract is considered as Fungistatic if the ratio MFC/MIC is higher than 1 and is considered as Fungicidal if this ratio is less than or equal to 1 [38].

### 2.7. Statistical Analysis

The data matrix with treatments, strains, doses, diameter, and the number of spores in the columns was subjected to ANOVA, fixed model in order to assess the effect of treatments, and doses on the growth of strains. It was carried out after checking the normality of logged data and the equality of variances. The structure test of averages SNK (Student-Newman-Keuls) was performed to identify the best treatment and the most effective dose. A treatment or dose is considered as effective when the mean diameter measured is the smallest. But there must be a significant difference between the terms of the factor considered. These tests were followed by the comparison test two by two of Bonferroni.

## 3. Results and Discussion

### 3.1. Presence of Scopoletin in Cassava Roots

**Figure 3:**
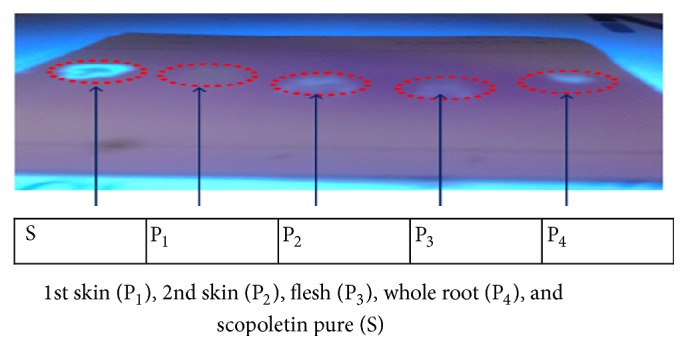
Qualitative assessment of scopoletin in the cassava roots.

The qualitative assessment of scopoletin in the cassava roots as described above in the methodology is presented in Figure 3. The extracts of various cassava root parts, namely, 1st skin (P_1_), 2nd skin (P_2_), flesh (P_3_), and whole root (P_4_), migrated at the same retention factor (RF) as reference scopoletin (S). Thus, we concluded that scopoletin was present in each part of the cassava root. These results are similar to those of Gnonlonfin et al. [39] who showed the presence of scopoletin in cassava roots produced in Benin. Furthermore, our results corroborated with those obtained by Tanaka et al. [40] and Buschmann et al. [30], who demonstrated the accumulation of scopoletin in the tuberous cassava roots during postharvest deterioration time.

### 3.2. Quantification of Scopoletin by HPLC

As previously described by Gnonlonfin et al. [41], high concentration (containing up to 242.5 mg/kg of dry matter) of scopoletin was found in cassava chips from BEN 86052. This justifies the use of cassava roots of this variety in the present study. The assay of scopoletin in the flour obtained from cassava chips with pure scopoletin showed a recovery rate of the method which was 98% in average with a linearity of *R*^2^ = 0,999 in the concentration interval between 200 ng/mL and 2000 ng/mL.

### 3.3. Antifungal Activity of Scopoletin on the Isolated Strains

**Figure 4:**
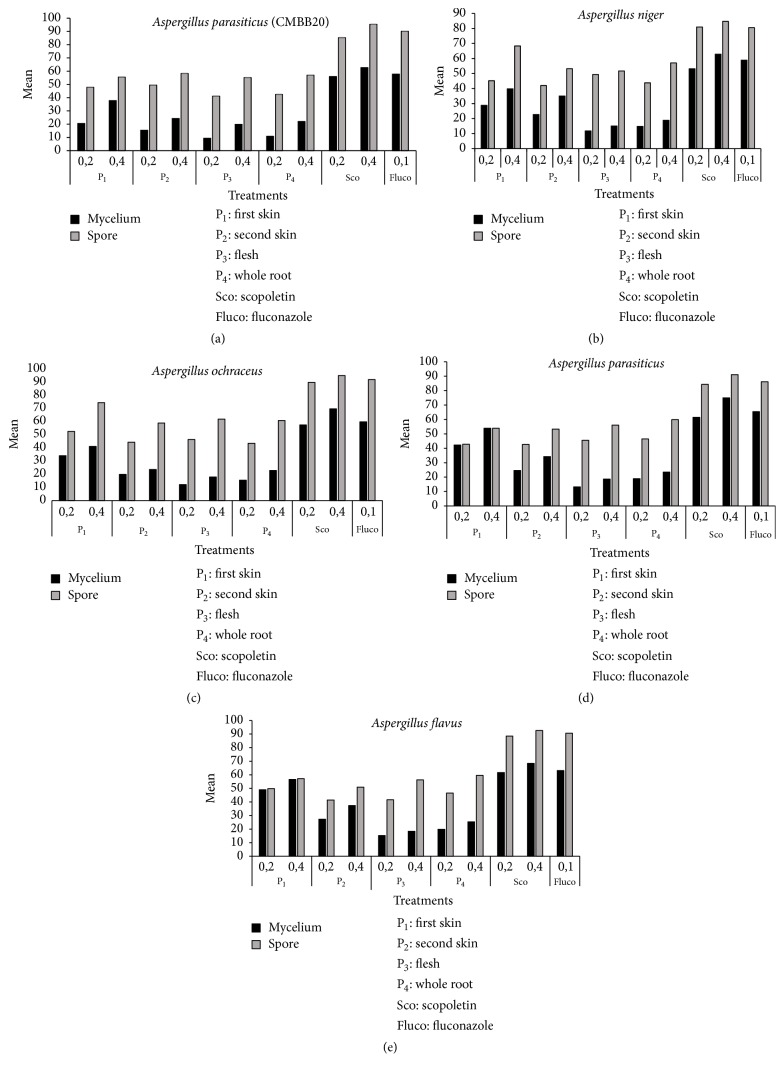
Inhibition percentages of mycelial growth and sporulation by* Aspergillus*.

**Figure 5:**
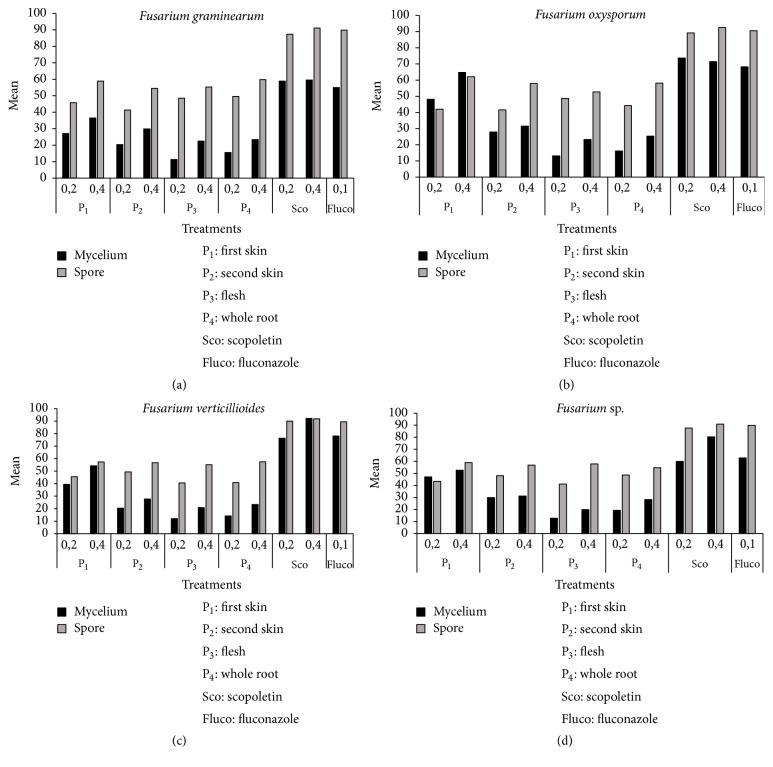
Inhibition percentages of mycelial growth and sporulation by* Fusarium*.

**Figure 6:**
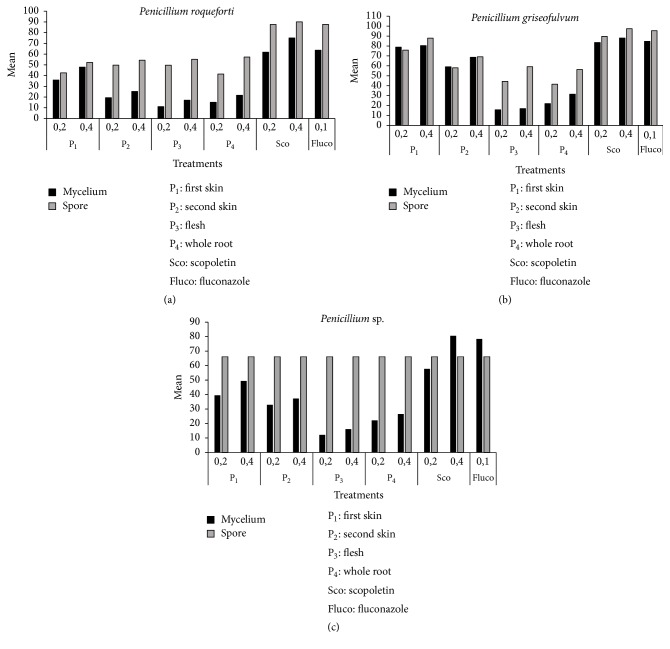
Inhibition percentages of mycelial growth and sporulation by* Penicillium*.

The percentages of inhibition of spores and mycelium growth of different fungi isolated from maize in storage are presented in Figures 4, 5, and 6. After five days of incubation at 28°C ± 2, we observed in all series of experiments compared to the controls a gradual decrease in the number of spores and mycelial diameter as the dose of the factor considered increases (Figures 4, 5, and 6). This was observed for all the series of tests. The analysis of variance performed on the diameter of the mycelial growth and on the number of spores was found highly significant (*p* < 0.001). The six treatments showed variable activity on fungal strains tested. Overall, only scopoletin extracts at the concentration of 0.4 mg/mL demonstrated the best percentages of inhibition for all strains tested. From the analysis of the results, it appears that the dose of 0.2 mg/mL was too small to significantly reduce mycelial growth. Pure scopoletin at the concentration of 0.4 mg/mL was the most active with the percentages of inhibition (PI) ranging from 59.50 to 92.14% for all strains. The use of fluconazole at a concentration of 0.1 mg/mL has shown PI ranging from 55.03 to 84.58%. These values obtained were lower than those of pure scopoletin. Scopoletin extracts from the cassava root also showed antifungal activity on the tested strains since they revealed inhibition rates ranging from 36.51 to 80.41% if they were from the first skin and between 24.33 and 68.56% if they were from the second skin and ranging from 18.86 to 28.18% if they were from the whole root. In fact, the diameters of the tested strains induced by their action were significantly lower than that showed under the action of pure scopoletin and fluconazole. Moreover, scopoletin extracted from the flesh of the cassava root showed, as in all the other cases, inhibition of mycelial growth compared to the negative control. However, these percentages of inhibition were lower than those of scopoletin extracted from the other components and this is regardless of the tested strain. These results confirm those of Gnonlonfin et al. [39] who proved the inhibitory effect of scopoletin on* A. flavus*. Furthermore, the inhibition of sporulation by the extracts varies according to the strains tested. The inhibitory effect of the extracts at the same concentration was more marked on sporulation than on the mycelial growth. The different treatments showed a percentage of inhibition of sporulation higher than 40% (Figures 4, 5, and 6). These inhibition percentages vary between 41.27% and 97.35%. Treatment with pure scopoletin was the most active on sporulation of fungal strains with inhibition percentages ranging from 90.11% to 97.35%. Scopoletin extracted from the first skin of cassava root inhibited sporulation of the strains tested, but these inhibitions' percentages were lower than those of pure scopoletin. As for treatment with scopoletin derived from the second skin of the cassava root, we also observed an inhibition of sporulation whatever the strain, but the recorded content was low when compared with the pure scopoletin and that extracted from the first skin (Figures 4, 5, and 6). For all strains, treatment with scopoletin extracted from the flesh revealed percentages of inhibition (51.70% to 59.16%) lower than those found for the treatment with scopoletin extracted from the skins and the whole root. From the results obtained, it appears that scopoletin was present in all parts of the cassava root. Furthermore, treatment with scopoletin extracted from the whole root revealed, as in other cases, an inhibition of sporulation, but the percentages found were lower than those of scopoletin extracted from the skins of cassava root. However, further studies are undergoing in our group to elucidate this preliminary observation.* P. griseofulvum* was more sensitive both to the inhibition of sporulation and to that of the mycelial growth. On the other hand, strains 2 and 6 were the least sensitive to the inhibition of mycelial growth and strains* A. parasiticus*,* F. oxysporum*, and* P. roqueforti* to the inhibition of sporulation. To the best of our knowledge this is the first time that the biological activity of scopoletin extracted from the cassava root components was evaluated on strains isolated from maize in storage. These results are similar to those obtain by Gómez-Vásquez et al. [42]. They have proved that scopoletin has Fungistatic and Fungicidal properties.

### 3.4. Determination of Minimum Inhibitory Concentrations of Scopoletin

The results of the determination of the MIC of scopoletin extracted from skins of cassava roots (first skin, second skin) are summarized in Table 2. From these results, it appears that the extract of scopoletin from the skins of cassava roots showed no inhibitory activity on the isolated fungi from maize in storage at the concentration of 0.00625 mg/mL. On the other hand, the pure scopoletin showed at this concentration antifungal activity. Above this concentration, the antifungal activity was observed on the 11 species of fungi isolated from maize and as well as the reference strain. The Minimum Fungicidal Concentration observed was between 0.0125 mg/mL and 0.1 mg/mL (Table 2). The analysis of the results shows that the isolated fungal strains of maize in storage and tested reference strain had varying sensitivities to the activity of the scopoletin extract. This could be justified by the fact that 9 out of 12 tested strains showed MFC between 0.0125 mg/mL and 0.1 mg/mL.* A. parasiticus*;* P. *sp.;* A. niger*;* A. ochraceus*;* F. verticillioides*;* F. *sp.;* P. griseofulvum*; and* A. parasiticus* (CMBB20) were the most sensitive strains. On the other hand, the following species:* A. flavus*;* F. oxysporum; F. graminearum*; and* P. roqueforti* were the least susceptible strains to the raw extract of scopoletin having revealed MFC/MIC ratios greater than or equal to 2. Thus, the fungal species* A. parasiticus* (CMBB20) and* P. griseofulvum* were the most susceptible because the concentrations of 0.0125 mg/mL and 0.025 mg/mL were enough to stop their growth. Moreover, the various MFC observed indicate that the extract of pure scopoletin demonstrated, at 0.00625 mg/mL, interesting antifungal activity on the tested fungal strains. Apart from the pure scopoletin, that obtained from the first skin of the cassava root has also shown an interesting antifungal activity. But the MFC observed were higher than that of pure scopoletin. This observation is probably due to the purity of the standard. In addition, the same antifungal activity was observed for scopoletin extracts from the second skin of the cassava root and the mixture of the first and second skins. This inhibitory effect could be explained by the fact that there was no difference between scopoletin extract from the first skin and the second skin. Overall, scopoletin, being a coumarin, shows an inhibitory effect on enzymes and the interactions with DNA [43, 44]. The antifungal activity of scopoletin from cassava root skins corroborates the studies of Rodriguez et al. [45] who showed that the antifungal activity is among different biological activities of scopoletin. As for Buschmann et al. [30], the accumulation of scopoletin in the chips is a defense response against invading microorganisms. Furthermore, the concentration of 0.1 mg/mL was not enough to stop the growth of* A. flavus*. This result is consistent with previous research conducted in Benin by Gnonlonfin et al. [46] and Gnonlonfin et al. [47]. These authors showed the absence of aflatoxin despite the presence of* A. flavus* in the samples of cassava chips. Similar results were also observed in Tanzania by Muzanila et al. [48], in Ghana by Wareing et al. [49], and in Nigeria by Jimoh and Kolapo [50]. The antifungal activity of the extract of the scopoletin from cassava root skins observed during this study will not only permit to value the waste from this plant but also to ensure the fungal control of maize in storage.

## 4. Conclusion

The study on controlling the growth of fungi in storage of maize by using scopoletin extracted from cassava roots produced in Benin revealed the presence of the latter in the various components of the cassava root. As a matter of fact, the various extracts showed antifungal activity on growth of the strains tested in vitro. The reference scopoletin at a concentration of 0.4 mg/mL was the most active. Among the extracts, only scopoletin from the first skin showed significant antifungal activity both for the inhibition of sporulation (52.29 and 87.91%) and for inhibition of mycelial growth (36.51–80.41%). The crude extracts from the manioc root skins showed, in vitro, a significant antifungal activity against the isolated maize fungi. This high bioactive power observed is attributed to the presence of scopoletin in the skins of the cassava root. Thus, the use of cassava root skins has a practical benefit for the fungal control of maize in storage.

## Figures and Tables

**Table 1:** Isolated strains from the maize in storage.

Number of strains	Species	Origin
(1)	*Penicillium *sp.	Storage maize
(2)	*Aspergillus niger*	
(3)	*Penicillium griseofulvum *	
(4)	*Aspergillus ochraceus*	
(5)	*Aspergillus flavus*	
(6)	*Fusarium graminearum*	
(7)	*Aspergillus parasiticus*	
(8)	*Fusarium oxysporum*	
(9)	*Fusarium *sp.	
(10)	*Fusarium verticillioides*	
(11)	*Penicillium roqueforti*	

(12)	*A. parasiticus (CMBB20)*	Reference

Be et al. [29].

**Table 2:** Minimum inhibitory concentrations of scopoletin extracted from skins of cassava roots (CMI and CMF).

Skins of cassava roots	Concentration (mg/mL)	*P. *sp.	*A. niger*	*P. griseofulvum*	*A. ochraceus*	*A. flavus*	*F. graminearum*	*A. parasiticus*	*F. oxysporum*	*F. *sp.	*F. verticillioides*	*P. roqueforti*	*A. parasiticus (CMBB20)*
1st skin (P_1_)	CMI	0.05	0	0.025	0.05	0.025	0.025	0.025	0.05	0.025	0.1	0.03	0.0125
	CMF	0.05	0	0.025	0.05	0.2	0.2	0.025	0.2	0.025	0.1	0.2	0.0125
	CMF/CMI	1	1	1	1	8	8	1	4	1	2	8	1

2nd skin (P_2_)	CMI	0.1	0.1	0.025	0.1	0.05	0.05	0.05	0.05	0.05	0.2	0.03	0.025
	CMF	0.1	0.1	0.025	0.1	0.1	0.2	0.05	0.2	0.05	0.2	0.2	0.025
	CMF/CMI	1	1	1	1	2	4	1	4	1	1	8	1

Mix of 1st skin and 2nd skin	CMI	0.1	0.1	0.025	0.1	0.05	0.05	0.05	0.05	0.05	0.2	0.03	0.025
	CMF	0.1	0.1	0.025	0.1	0.1	0.2	0.05	0.2	0.05	0.2	0.2	0.025
	CMF/CMI	1	1	1	1	2	4	1	4	1	1	8	1

Pure scopoletin	CMI	0.025	0	0.0125	0.05	0.025	0.025	0.013	0.01	0.013	0.1	0.03	0.00625
	CMF	0.025	0	0.0125	0.05	0.05	0.1	0.013	0.05	0.013	0.1	0.1	0.00625
	CMF/CMI	1	1	1	1	2	4	1	4	1	1	4	1

*P*: *Penicillium*; *F*: *Fusarium*; and *A*: *Aspergillus*.
